# Preoperative Transcranial Direct Current Stimulation in Glioma Patients: A Proof of Concept Pilot Study

**DOI:** 10.3389/fneur.2020.593950

**Published:** 2020-11-19

**Authors:** Stefan Lang, Liu Shi Gan, Cael McLennan, Adam Kirton, Oury Monchi, John J. P. Kelly

**Affiliations:** ^1^Hotchkiss Brain Institute, University of Calgary, Calgary, AB, Canada; ^2^Department of Clinical Neurosciences, University of Calgary, Calgary, AB, Canada; ^3^Non-invasive Neurostimulation Network, University of Calgary, Calgary, AB, Canada; ^4^Charbonneau Cancer Institute, University of Calgary, Calgary, AB, Canada

**Keywords:** glioma, plasticity, transcranial direct current stimulation, functional connectivity, sensorimotor network, finite element model

## Abstract

**Background:** Transcranial direct current stimulation (tDCS) has been used extensively in patient populations to facilitate motor network plasticity. However, it has not been studied in patients with brain tumors. We aimed to determine the feasibility of a preoperative motor training and tDCS intervention in patients with glioma. In an exploratory manner, we assessed changes in motor network connectivity following this intervention and related these changes to predicted electrical field strength from the stimulated motor cortex.

**Methods:** Patients with left-sided glioma (n=8) were recruited in an open label proof of concept pilot trial and participated in four consecutive days of motor training combined with tDCS. The motor training consisted of a 60-min period where the subject learned to play the piano with their right hand. Concurrently, they received 40 min of 2 mA anodal tDCS of the left motor cortex. Patients underwent task and resting state fMRI before and after this intervention. Changes in both the connectivity of primary motor cortex (M1) and general connectivity across the brain were assessed. Patient specific finite element models were created and the predicted electrical field (EF) resulting from stimulation was computed. The magnitude of the EF was extracted from left M1 and correlated to the observed changes in functional connectivity.

**Results:** There were no adverse events and all subjects successfully completed the study protocol. Left M1 increased both local and global connectivity. Voxel-wide measures, not constrained by a specific region, revealed increased global connectivity of the frontal pole and decreased global connectivity of the supplementary motor area. The magnitude of EF applied to the left M1 correlated with changes in global connectivity of the right M1.

**Conclusion:** In this proof of concept pilot study, we demonstrate for the first time that tDCS appears to be feasible in glioma patients. In our exploratory analysis, we show preoperative motor training combined with tDCS may alter sensorimotor network connectivity. Patient specific modeling of EF in the presence of tumor may contribute to understanding the dose-response relationship of this intervention. Overall, this suggests the possibility of modulating neural networks in glioma patients.

## Introduction

Transcranial direct current stimulation (tDCS) is a non-invasive neuromodulation technique which passes a low amplitude electrical current into the brain. While most of the current is shunted by the scalp (1), multiple studies suggest a biologically relevant portion reaches the brain (2, 3). The effect of this is complex but may result in a polarity dependent modulation of the resting membrane potential (4), an alteration of spontaneous firing rates (4–6), a change in the local excitatory/inhibitory balance (7), an alteration of neuronal oscillatory patterns (8), and a change in the synchronization of activity in distant brain regions (9–12). This technique has been shown to facilitate motor learning (13) and cortical plasticity in healthy subjects, as well as in disease states (14). While this technique has been used extensively in the motor rehabilitation and neuropsychiatric literature (15–17), it has never been investigated in the context of brain tumors. Gliomas are the most common primary brain tumor and are associated with high rates of neurological comorbidities, including motor and language deficits, as well as neuropsychiatric conditions (18–22). This patient population may therefore benefit from investigational use of tDCS. One unique opportunity arises in the context of “eloquent” (primary motor/language) cortex tumors. Mounting evidence suggests that an aggressive surgical resection improves overall survival in glioma patients (23, 24). However, tumors located near eloquent cortex represent a particularly difficult challenge due to the high rates of neurological morbidity following surgical resection. Specifically, the risk of permanent neurological deficit reaches 40% when motor cortex lesions are resected (25). Therefore, location within these critical regions is a major limitation toward the gold standard of maximal resection. Overcoming this difficult problem will require novel and innovative strategies. One proposed strategy has arisen from the observation that patients with tumor in close proximity to these critical regions may occasionally have minimal symptoms compared to that which may be expected based on size and location alone. In these patients, it is thought that the slow growing nature of the lesion has resulted in a dramatic reorganization of cortical structure and function, such that other regions of the brain have become involved in the implementation of the critical functions (26). This remarkable plasticity can allow for aggressive resection within classically eloquent regions (27). This exemplifies the fact that critical cortical regions, such as primary motor cortex, can be removed if their function has been redistributed to alternative regions of brain. Surgeons have used this phenomenon to achieve greater resection of tumor tissue around motor and language eloquent areas. For example, Gil Robles et al. (28) performed intraoperative cortical stimulation in multiple staged surgeries of low grade glioma of motor cortex to show that increased extent of resection was possible during the second surgery. This was proposed to be due to redistribution of functional tissue away from the residual tumor. This idea was taken further in a study which attempted to facilitate this functional reorganization in-between staged surgeries (29). In this pilot study, surgeons implanted a grid of electrodes over residual tumor which contained functional tissue. These electrodes provided continuous cortical stimulation, that when combined with a physiotherapy routine, presumably facilitated the redistribution of function out of these regions without a corresponding decrease in motor ability. This allowed for more extensive resection during a second surgery. While promising, this study utilized invasive cortical electrode implantation which was associated with significant complications (infection). Further, the mechanism of this effect was not investigated. Based on these ideas, we aimed to investigate, for the first time, the use of tDCS in glioma patients with the goal of neuromodulation. Importantly, tDCS has been shown to increase functional connectivity in the sensorimotor network (30). Functional connectivity of the sensorimotor network is related to motor performance in glioma patients (31) with increased connectivity related to better performance. Therefore, we attempted to facilitate plasticity of the sensorimotor network in patients during the preoperative period. To achieve this objective, we used a motor training program combined with tDCS. Functional connectivity analyses of BOLD MRI data were used to measure changes in the sensorimotor network, and patient specific computational modeling was used to relate any changes to the magnitude of the applied electrical field (EF). We hypothesize that this intervention will facilitate cortical plasticity, measured by increased connectivity of the sensorimotor network. To examine this, we first assessed the interhemispheric primary motor cortex (M1) connection, followed by an assessment of the global and local connectivity of M1. We were also interested in examining more general connectivity changes which may not be limited to the motor network and required no assumptions about location. To accomplish this, we assessed voxel-wide measures of global and local connectivity. Overall, this research has the potential of leading to novel clinical strategies for treating tumors within or near eloquent cortex.

## Methods

### Subjects

**Figure 1:**
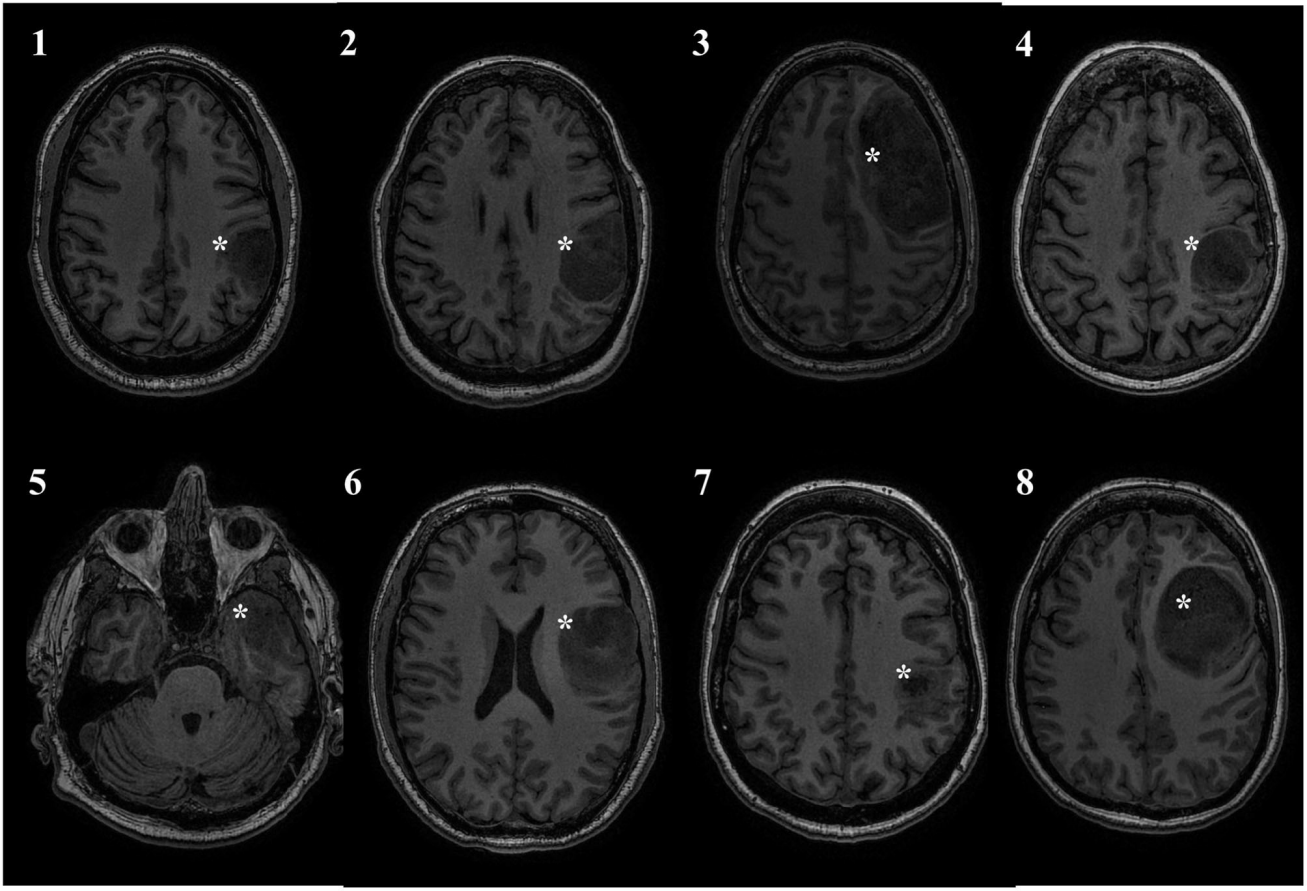
Tumor location. All patients had left sided tumors, and 7/8 were in close proximity to the central sulcus. Tumor marked with a white asterisk.

This study was approved by the Conjoint Health Research Ethics Board of the University of Calgary, and all patients provided informed consent. Over a period of 2 years, eight adult patients (mean age = 46.6 ± 15.46) with left-sided diffuse gliomas primarily of the frontal and parietal regions were recruited from the University of Calgary surgical neurooncology clinic. Inclusion criteria included the presence of a presumed glioma in an ambulatory patient and the lack of significant neurological deficits precluding participation in the training program. With the exception of one patient, the tumor was located in close proximity to the precentral gyrus. Exclusion criteria included patients requiring emergent or urgent surgery, bilateral or right-sided tumor involvement, excessive midline shift, excessive peri-tumoral edema, and poorly controlled seizure activity. Exclusion criteria for the study also included contraindications to MR imaging (e.g., claustrophobia, implanted ferromagnetic devices, pregnancy). Demographic and tumor details are displayed in Table 1. Figure 1 displays an axial T1WI in the plane of each subject's tumor.

**Table 1:** Demographic and tumor related data.

**Patient**	**Age**	**Gender**	**Hand**	**Tumor location**	**Presenting symptom**	**Tumor grade**	**Genetics**
1	46	M	L	Left post-central	Seizure	Grade III Astrocytoma	IDH mt, ATRX loss, MGMT methylated
2	63	F	R	Left post-central	Seizure	GBM	IDH WT, ATRX retained, p53 positive, MGMT unmethylated
3	31	M	R	Left frontal	Headache	Grade III Astrocytoma	IDH1 mt, ATRX loss, MGMT methylated, p53 positive
4	64	F	R	Left post-central	Word finding difficulty, right hand sensory deficit	GBM	IDH WT, ATRX retained, MGMT methylated
5	57	M	R	Left temporal	Seizure	Oligodendroglioma	IDH mt, 1p/19q codeletion
6	32	M	R	Left frontal	Seizure	GBM	IDH mutant, ATRX loss, MGMT methylated
7	55	F	R	Left frontal	Face numbness, right hand incoordination and dysarthria	GBM	IDH WT, p53 positive, MGMT methylated
8	25	F	R	Left frontal	Seizure	Grade II Astrocytoma	IDH mt, ATRX loss, MGMT methylated

### tDCS and Motor Training

**Figure 2:**
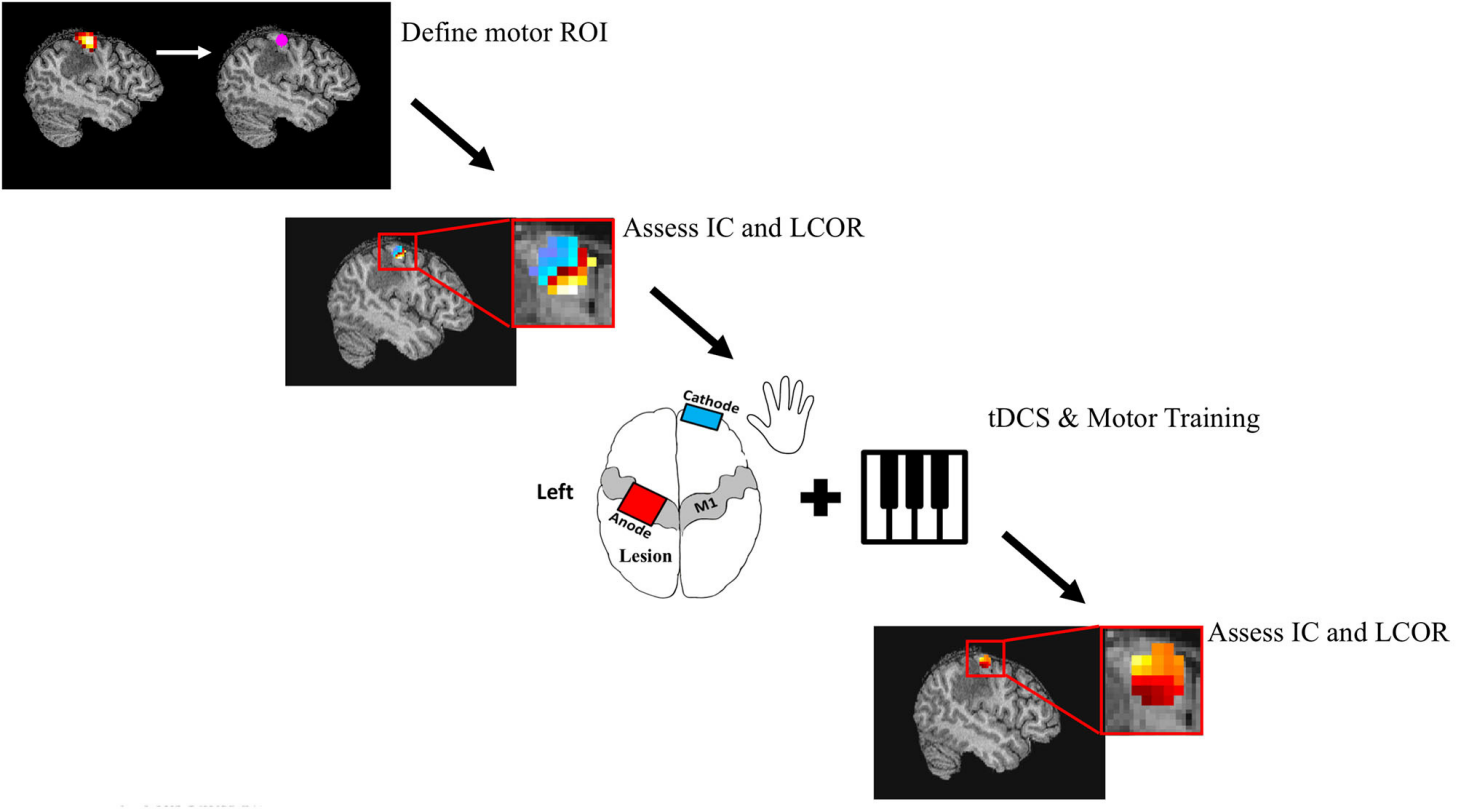
Study Protocol. Patients underwent both task and resting state fMRI. Task fMRI was used to determine subject specific ROI's in the primary motor cortex. Resting state fMRI was used to calculate the global (Intrinsic Connectivity; IC) and local (Integrated local correlation; LCOR) connectivity of these M1 seeds. Patients then underwent 4 consecutive days of motor training and tDCS. The motor task consisted of a total of 60 min of individualized piano training, while a total of 40 min of 2 mA anodal tDCS was applied over the left motor cortex. Finally, global and local connectivity of M1 was calculated 24 h following the intervention.

On four consecutive days, patients participated in motor training sessions combined with tDCS. tDCS was applied with the anode positioned over the left primary motor cortex and the cathode over the contralateral supraorbital area. The primary motor cortex was localized using the 10–20 Electroencephalography Electrode System (C3). All patients received active stimulation. Anodal tDCS was delivered through 35 cm^2^ saline-soaked sponge electrodes using a DC Stimulator (Soterix Medical Inc., New York, USA). Current was ramped up to 2 mA over 30 s and maintained for 20 min. Immediately following application of the tDCS, subjects started training on a unilateral, right handed music rhythm task of manual dexterity. In this task, subjects were given personalized piano playing instructions over the course of 30 min. This piano playing task was chosen because it is highly engaging for the subjects and requires focused attention. The first 20 min of this was done with concurrent stimulation. Once 30 min of training had occurred, the entire stimulation and training procedure was repeated. This resulted in a total of 40 min of stimulation and 60 min of training per day. Patients were piano naive, with the exception of one who had some experience as a child. The study protocol is displayed in Figure 2.

### MRI Acquisition

All patients underwent both resting-state and task-based fMRI before and after the motor training/tDCS intervention. Task fMRI consisted of a hand clenching task, designed to elicit activation of the primary motor cortex. This task is routinely administered as part of a pre-surgical work-up to map motor regions. Participants open and close their hand in time with a visual cue, with each run alternating blocks of task and rest. This was performed unimanually with separate runs for each hand. The order of these runs were randomized, and each run was performed for a period of ~4 min. All MRI data was acquired using a 3 Tesla GE Discovery MR750 whole body scanner with a receive-only 12-channel phased-array head coil (GE Healthcare, Waukesha, WI). Each participant's head was immobilized using foam cushioning, and participants had the option to terminate the study at any time during the scan using a squeeze ball placed in their hand. Resting-state fMRI was collected for two runs of 5 min using a gradient-recalled echo, echo planar imaging sequence (voxel dimensions 3.75 × 3.75 × 4 mm, 30 slices, 4-mm slice thickness, 64 × 64 matrix, TE = 30 ms, TR = 2,000 ms, flip angle = 65 degrees). Subjects were instructed to look at a fixation cross, let their mind wander freely, and to not fall asleep. T1-weighted multi-slice spoiled gradient (30 slices, 4-mm thickness, 128 × 128 matrix, minimum TE, TR = 150 ms, flip angle = 18 degrees) and 3D magnetization-prepared gradient-echo sequences (1.3 mm slices, 384 × 256 × 112 matrix, preparation time = 500 ms, minimum TE, TR = 8.9 ms, flip angle = 11 degrees) were collected for anatomical registration of the fMRI data. Task fMRI was also collected using a gradient-recalled echo, echo planar imaging sequence (voxel dimensions 3.75 × 3.74 × 4 mm, 28 slices, 4 mm slice thickness, 64 × 64 matrix, TE = 30 ms, TR = 1.5 s, flip angle = 65 degrees). Two scans were completed for each hand, for a total of four task fMRI runs.

### Task fMRI Analysis

Task-fMRI data were analyzed to identify subject-specific seeds to be used in the subsequent ROI connectivity analyses. Images were preprocessed using SPM 12 software. Preprocessing steps included realignment, motion correction, co-registration of functional and structural images, non-linear normalization to MNI space, and smoothing using a 6-mm FWHM Gaussian kernel. A time series general linear model analysis was performed on each patients' data, contrasting the motor task trial blocks with the rest blocks. The peak activation voxel from this contrast was used as the center of a 6-mm spherical ROI. This was performed separately for the data from both hands, resulting in two 6 mm spherical ROIs centered over bilateral primary motor cortex. In one subject, the peak activation voxel was located in the cerebellum and therefore the second highest voxel was chosen, which was located in the expected region of primary motor cortex. MNI coordinates for each subjects M1 ROI's can be found in Supplementary Table 1.

### Resting State fMRI Preprocessing

Images were preprocessed and analyzed using the SPM toolbox Conn (32) (https://www.nitrc.org/projects/conn). Briefly, functional images underwent realignment, motion correction, slice-time correction, co-registration to high resolution structural images, and non-linear normalization to MNI space. The structural images were segmented into gray matter, white matter and CSF. Quality assurance, to detect outliers in motion and global signal intensity, was performed using the software *art* as implemented in Conn (https://www.nitrc.org/projects/artifact_detect). Outliers were included as regressors in the first level analysis, along with motion parameters and their first temporal derivatives. Physiological and other sources of noise from the white matter and CSF signal were estimated using the aCompcor method (32–34) and removed with the other covariates. The residual BOLD time series was high pass filtered at 0.009 Hz.

### First Level Analysis

#### M1 Interhemispheric Connectivity

In order to specifically assess the interhemispheric connectivity within the sensorimotor network, we extracted the average residual BOLD time course (during the resting-state scans) from individualized seeds placed within left (stimulated) and right (non-stimulated) M1 regions. The Fisher Z transformed Pearson correlation coefficient was calculated between these two time courses.

#### M1 Global Connectivity

To assess global connectivity changes of M1, we computed a measure of network centrality known as intrinsic connectivity (IC) (35). This measure is characterized by the strength of connectivity between a given voxel and the rest of the brain. It is defined as the root mean square of correlation coefficients between each voxel and all the voxels in the brain.

$$\begin{array}{l} IC(x)=\frac{\surd \sum_{y\in M}r{\left(x,y\right)}^{2}}{N} \end{array}$$

Where *IC(x)* = Intrinsic Connectivity at voxel *x*; *r(x,y)* = correlation coefficient between voxels *x* and *y*; and *N* = number of voxels. Subject specific dimensionality reduction of the voxel to voxel correlation matrices to 64 components was initially performed using singular value decomposition, followed by calculation of IC. These values are subsequently normalized. The resultant IC value was averaged over each voxel within the M1 seeds to derive a measure of M1 global connectivity.

#### M1 Local Connectivity

To assess local connectivity changes of M1, we next computed a measure of local coherence known as the integrated local correlation (LCOR) (36). LCOR is defined as the average of correlation coefficients between each individual voxel and a region of neighboring voxels. A full width half maximum kernel of 8 mm was used as a local weighting function.

$$\begin{array}{l} LCOR\left(x\right)=\frac{\sum_{y\epsilon M}w\left(x-y\right)r\left(x,y\right)}{\sum_{y\epsilon M}w\left(x-y\right)}\\ \;w\left(z\right)=e^{\frac{-{\left|z\right|}^{2}}{{2\sigma }^{2}}} \end{array}$$

Where *LCOR(x)* = local correlation at voxel *x*; *r(x,y)* = correlation coefficient between voxels *x* and *y*; and *w(z)* = isotropic Gaussian weighting function. The LCOR measure was averaged over each voxel within the M1 seeds to derive a measure of M1 local connectivity.

#### M1 to Whole Brain Connectivity

To further assess the connectivity of M1, we performed a ROI to whole brain analysis using the same M1 seeds derived for each subject from the task fMRI analysis. The Fisher transformed Pearson correlation between the average BOLD signal from the left and right ROI and the signal from each voxel in the brain was calculated.

#### Global and Local Connectivity Across the Whole Brain

To determine if connectivity changes were occurring in regions of the brain outside of M1, we assessed changes in IC and LCOR on a voxel-wide manner, without restricting the analysis to a specific ROI.

### Second Level Analysis and Statistics

The Shapiro-Wilk test was used to assess for normality of data. Changes in interhemispheric connectivity, M1 global connectivity, and M1 local connectivity were assessed with a one-tailed paired *t*-test, considering our hypothesis of increased M1 connectivity. Significance was determined at *p* < 0.05. Voxel-wide second level analyses (M1 to whole brain and voxel-wide global & local connectivity) were implemented in Conn using the general linear model and the likelihood ratio test to evaluate model parameters. Clusters were thresholded with a significance of *p* < 0.005 at the voxel level and *p* < 0.05 corrected for multiple comparisons with the false discovery rate at the cluster level. Linear regression was used to relate the change in global and local connectivity from right and left M1 with the average electrical field magnitude extracted from the left M1 ROI. Significance was set at *p* < 0.05.

### Patient Specific Electrical Field Modeling

**Figure 3:**
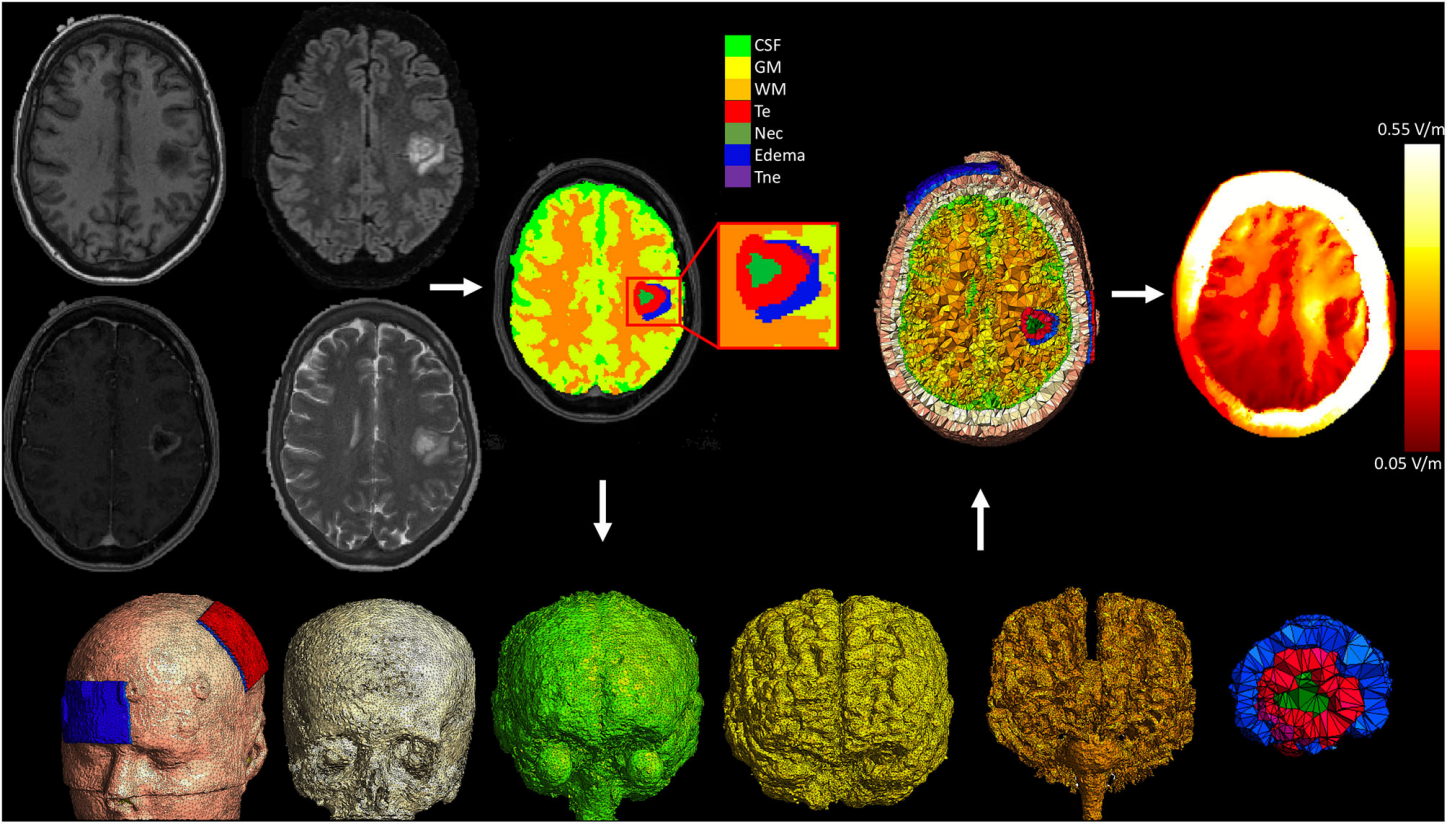
Pipeline for patient specific electric field modeling. Each subject underwent T1WI, T2WI, T1WI with gadolinium, and FLAIR imaging as part of their routine clinical care. These images were used to segment the head, brain, and tumor into component tissue classes. A modified version of ROAST was then used to mesh the volumes and solve the finite element model. CSF, cerebrospinal fluid; GM, gray matter; WM, white matter; Te, enhancing tumor; Nec, necrosis; Tne, non-enhancing tumor.

We performed patient specific computational modeling of the electric field resulting from the tDCS intervention, taking into consideration the anatomy and tissue components of the tumor. Methodology was similar to our previous work modeling electric fields in glioma patients (37). Briefly, to perform detailed tumor segmentation, we used four MRI sequences (T1WI, T2WI, T1WI with Gadolinium and FLAIR) acquired during the routine clinical care of each patient. Using these scans as input, each subjects brain tumor was segmented into component tissues classes using an automated segmentation software [BraTumIA (38)], followed by manual correction. The tissues classes included in the model included non-enhancing tumor, enhancing tumor, necrosis, and edema. Finite element models were then created using a modified version of the Realistic vOlumetric Approach to Simulate Transcranial electrical stimulation (ROAST) pipeline (39). ROAST uses SPM12 to segment the entire head and neck and combines this with a post-processing routine to ensure continuity of CSF. A tetrahedral volume mesh is then created with iso2mesh (40), and the Laplace equation for voltage distribution is solved using getDP (41). Custom MATLAB scripts were integrated into this pipeline to allow for the addition of the tumor component masks, each with a unique conductivity. Enhancing and non-enhancing tumor were assigned conductivity values of 0.170 S/m and 0.332 S/m respectively (42, 43). Necrosis and edema tissues were assigned conductivities of 1.0 S/m, and 1.185 S/m (44, 45). The default values for conductivity of healthy tissue classes in the ROAST pipeline were used (white matter: 0.126 S/m; gray matter: 0.276 S/m; cerebrospinal fluid: 1.65 S/m; bone: 0.01 S/m; skin: 0.465 S/m; air: 2.5^−14^ S/m; gel: 0.3 S/m; electrode: 5.9^7^ S/m). Electrodes (5 × 7 cm^2^) were placed at C3 and FP1, simulating the anodal M1 tDCS configuration performed in the study. The average electric field strength (the vector norm of the electric field) was extracted from the left M1 ROI and used in the regression with connectivity values. Figure 3 displays the pipeline for a representative subject.

## Results

### tDCS and Motor Training Compliance and Tolerability

All patients were examined clinically, and no motor deficits were noted prior to enrollment. Five presented with a new onset seizure, though these were under control prior to initiating the experimental paradigm. One patient was found to have sensory disturbance in the right hand, while another had minor complaints of incoordination. One subject had subjective language complaints. The clinical exam was otherwise unremarkable for all other subjects. All patients, with the exception of one, completed the study visits as designed. In each case, the follow-up fMRI was performed 24 h after the final training session. In one patient, three of the four intervention days were completed due to subject preference, and the follow-up MRI was performed 48 h following the last training session. All subjects tolerated the stimulation and there were no adverse effects observed. No seizures were noted during the experimental period. Minor tingling and itching sensations were reported by all subjects, consistent with the vast tDCS safety literature (46). All patients subjectively improved motor performance on the task.

### M1 Interhemispheric Connectivity

All connectivity and electrical field data were normally distributed as determined by the Shapiro-Wilk test. Patients on average showed an increase in interhemispheric connectivity following the intervention (0.095 ± 0.16). However, this difference did not reach threshold for statistical significance [*t*_(7)_ = 1.62, *p* = 0.0743].

### M1 Global Connectivity

**Figure 4:**
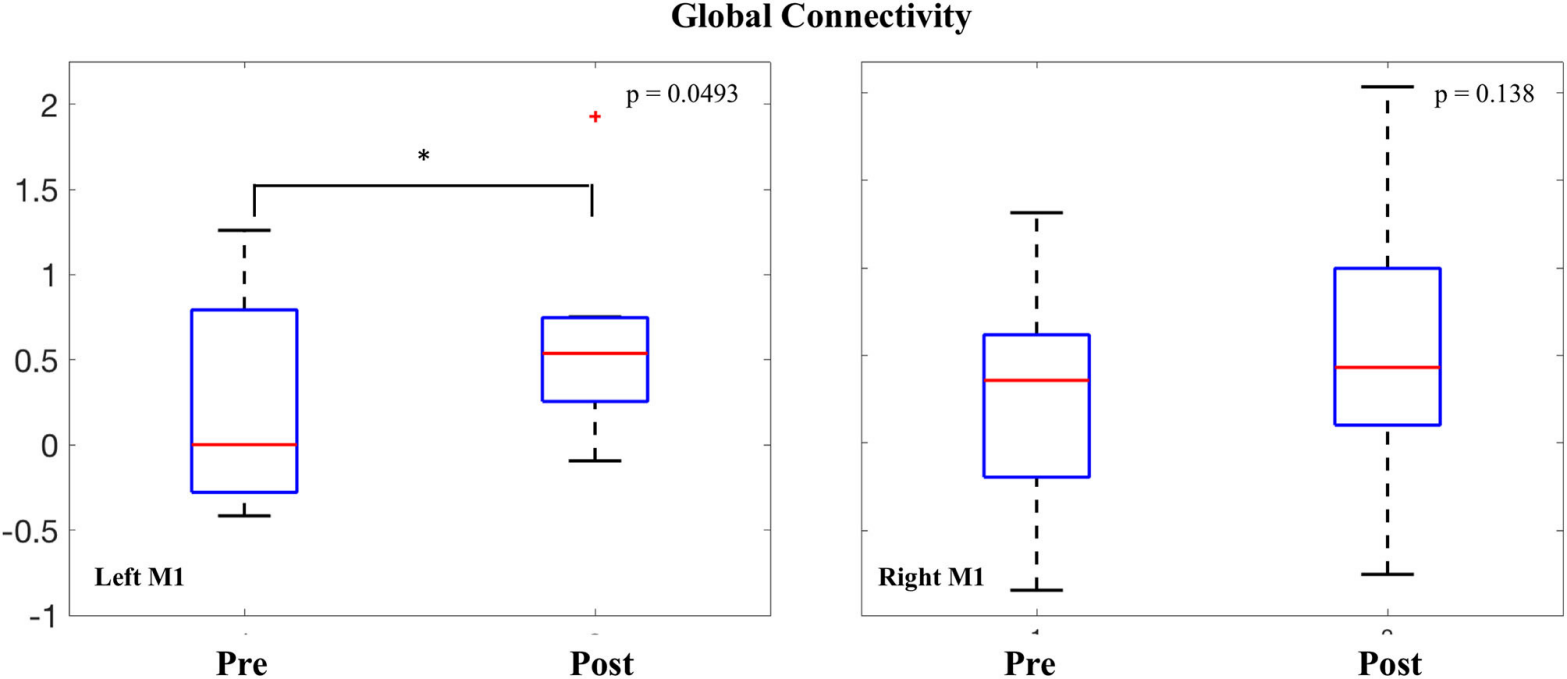
Change in global connectivity of M1.

Intrinsic connectivity values were averaged within both the right and left M1 seeds and compared before and after the intervention. In the left (stimulated) M1, a significant increase was observed in global connectivity [0.380 ± 0.56; *t*_(7)_ = 1.90, *p* = 0.0493]. In the right (non-stimulated) M1, no difference was observed in global connectivity values before and after the intervention [0.290 ± 0.69; *t*_(7)_ = 1.18, *p* = 0.138] (Figure 4).

###  M1 Local Connectivity

**Figure 5:**
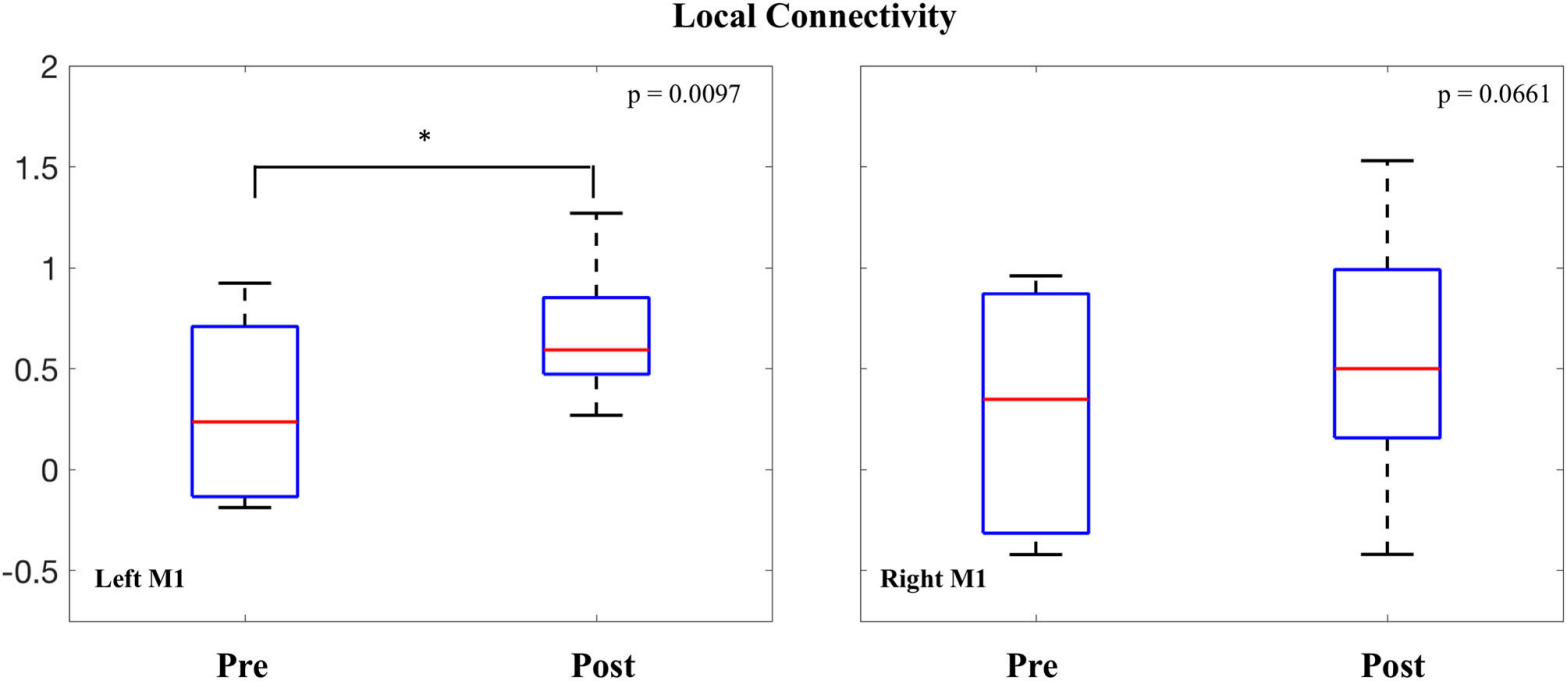
Change in local connectivity of M1.

LCOR values were also averaged within both the right and left M1 seeds and compared before and after the intervention. In the left M1, a significant increase was observed in local connectivity [0.377 ± 0.35; *t*_(7)_ = 3.02, *p* = 0.0097]. In the right M1, the change in average local connectivity did not reach threshold for statistical significance [0.257 ± 0.43; *t*_(7)_ = 1.7, *p* = 0.0661] (Figure 5).

### M1 to Whole Brain Connectivity

To further assess the change in connectivity of M1, the time course of individualized seeds placed in left and right M1 were correlated with the time course from each voxel across the entire brain and compared before and after the intervention. No significant clusters were identified from either the left or right M1 seed.

### Global and Local Connectivity Across the Whole Brain

**Figure 6:**
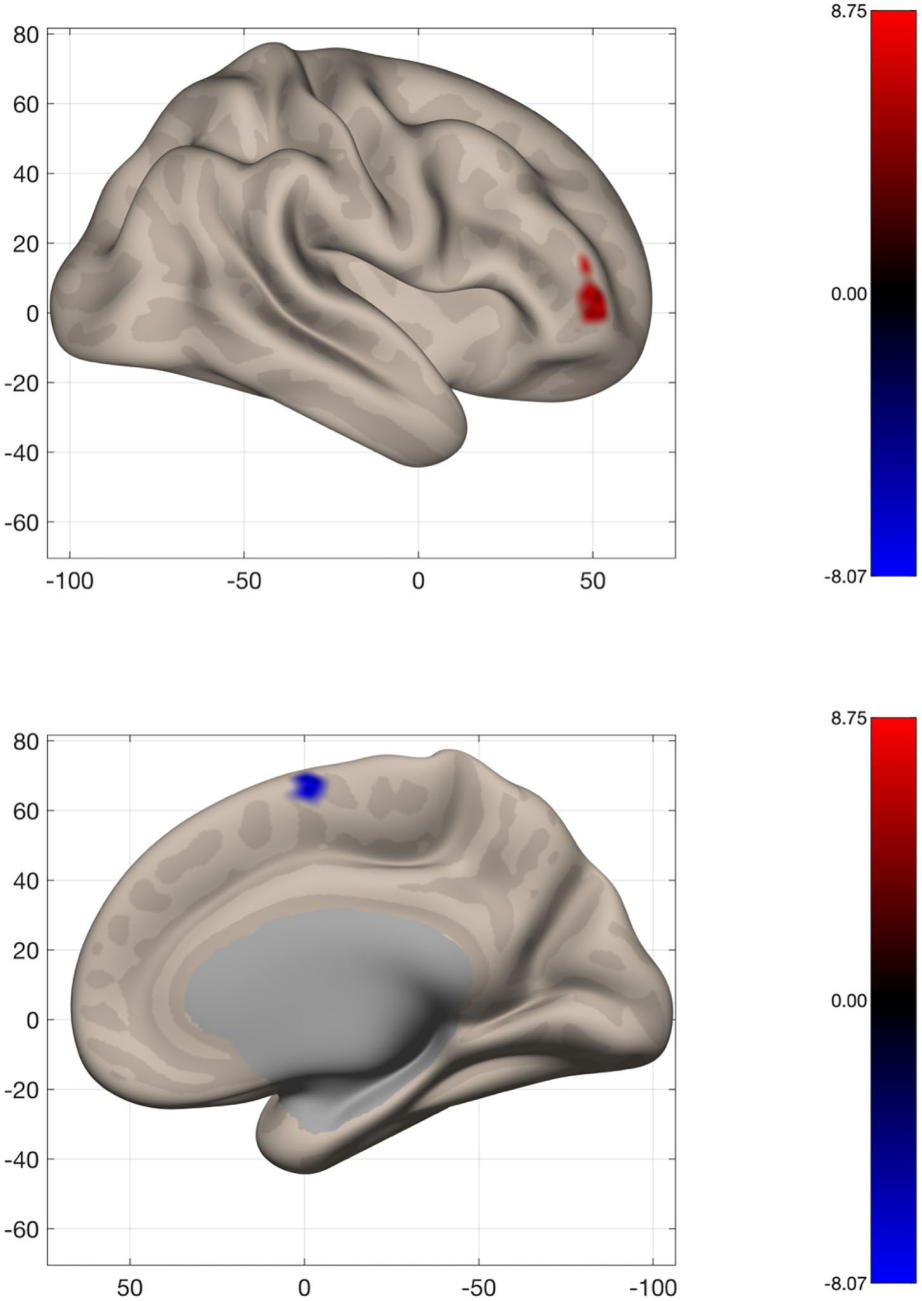
Voxel-wide global connectivity alterations. Significant clusters are seen in the right frontal pole (increased) and supplementary motor area (decreased). Color bar represents T-score.

To assess changes in connectivity which may not be limited to the sensorimotor network, we calculated IC and LCOR for every voxel in the brain and compared these values before and after the intervention. Following the intervention, patients had less global connectivity in a cluster spanning the supplementary motor cortex, while they showed increased connectivity in a cluster located in the right frontal pole (Figure 6). When assessed across the entire brain, no significant clusters of LCOR were observed. MNI coordinates and statistics of the significant clusters are displayed in Table 2.

**Table 2:** Cluster location and statistics from voxel-wide analysis of Intrinsic Connectivity (IC).

**Analysis**	**Cluster**	**MNI (x, y, z)**	**Size (voxels)**	**Peak *p*-value**	**Anatomical location**
**IC**	1	42, 46, 06	76	0.000032	Right frontal pole
	2	06, −02, 62	51	0.000044	Right Supplementary Motor Area

### Patient Specific Electric Field Modeling

**Figure 7:**
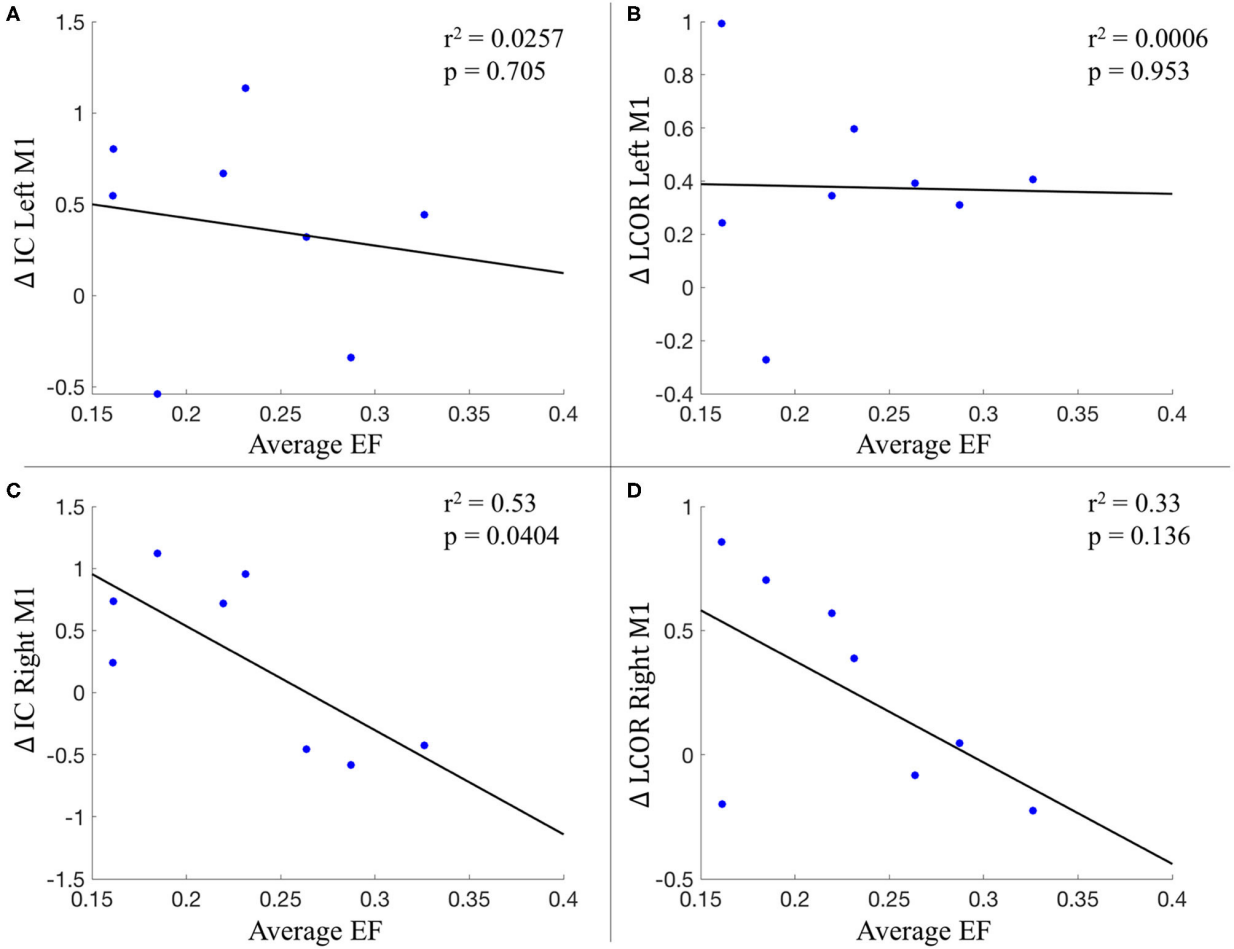
Relationship between the average electric field from left M1 and connectivity changes**. (A)** Change in IC of left M1; **(B)** Change in LCOR of left M1; **(C)** Change in IC of right M1; **(D)** Change in LCOR of right M1. IC, intrinsic connectivity; LCOR, Integrated local correlation; EF, electric field.

The average electrical field strength in the brain was 0.196 ± 0.02 V/m (range 0.17–0.23 V/m), while the average EF from the left M1 ROI was 0.229 ± 0.06 V/m (range 0.16–0.33 V/m). The correlation between the average strength of the EF in left M1 and the change in global (*r*^2^ = 0.0257; *p* = 0.705) and local connectivity (*r*^2^ = 0.0006; *p* = 0.953) also from left M1 did not reach statistical significance. This was also true for the correlation between the change in local connectivity from right M1 with the EF magnitude from left M1 (*r*^2^ = 0.33; *p* = 0.136). However, there was a significant correlation between the EF magnitude in left M1 and the change in global connectivity from the right M1 (*r*^2^ = 0.53; *p* = 0.0404) (Figure 7).

## Discussion

In this proof of principle pilot study, we show for the first time that tDCS is feasible in glioma patients. Further, we demonstrate that motor training, combined with tDCS, may alter sensorimotor network connectivity in this patient population. Patients with left-sided diffuse glioma (primarily of the frontal and parietal lobes and in proximity to the central sulcus) underwent repeated motor training using a piano playing task, while anodal tDCS was applied to the motor cortex. Functional MRI was performed before and after this intervention, and changes in brain connectivity were assessed. Connectivity of the sensorimotor network, using individualized ROI's within the primary motor cortex, was computed. We assessed the interhemispheric connection between bilateral M1 as well as the global and local connectivity of M1 more broadly. We then performed voxel-wide analyses to assess for connectivity changes which did not depend on *a priori* ROIs. Interhemispheric connectivity did not change as a result of our intervention. However, the functional connectivity of M1 was altered more broadly. Patients had both increased global and local connectivity of M1 following the intervention. The seed to whole brain analysis did not show significant clusters, suggesting the increased global and local connectivity observed in M1 was not due to any particular connection. We then performed two data driven analyses in order to assess for connectivity changes occurring across the whole brain. Global connectivity increases were seen in the right frontal pole, while decreases were seen in the supplementary motor cortex. Taken together, the motor training/tDCS intervention may have resulted in increased global and local connectivity of M1, which did not appear to be due to a particular connection, while other regions showed altered global connectivity. Speculatively, decreased global connectivity of the pre-SMA may result from motor learning, as this region has been shown to decrease its contribution to motor tasks with increased practice (47). Increased right frontal global connectivity may have occurred from the cognitive demands of learning action-outcome associations required for successful task performance (48).

Patient specific models of the applied electrical field were created, taking into consideration the anatomy and conductivity of tumor tissue components. The average field strength from the stimulated (left) M1 was extracted and related to the connectivity changes of both the right and left M1. Surprisingly, there was no relationship between the applied EF and connectivity changes of the ipsilateral M1, while there appeared to be an inverse relationship with connectivity changes in the contralateral hemisphere. It is uncertain what this relationship means, though it suggests a possible dose-dependent effect of tDCS on connectivity changes. Speculatively, it suggests an inhibitory effect of the stimulation on contralateral plasticity. Further work is needed to try to elucidate this relationship.

The combination of tDCS and motor training to facilitate cortical plasticity has been studied extensively in the motor rehabilitation literature. The highest volume of data comes from adult stroke rehabilitation, where pooled analyses suggest beneficial motor effects (49), likely in a dose dependant manner (16). The novelty of our study comes from applying these insights for the first time to glioma patients and attempting to facilitate an on-going plasticity process before a planned insult occurs. The idea of attempting to prevent deficits (rather than treat deficits) derives from the concept of prehabilitation, which has gained traction in recent years. It represents a paradigm change away from the reactive model of healthcare toward a proactive approach which engages patients in their care (50). Prehabilitation of the brain presupposes that changes in functional networks are occurring that may confer resiliency against insult. Our results support the idea that periods of prehabilitation can result in connectivity changes of cortical networks, though no conclusions can be made regarding the clinical impact of these changes. Speculatively, we suggest that increased connectivity of the sensorimotor network may increase “motor reserve,” a testable hypothesis which is critical to the clinical translation of this work. This idea stems from the concept of “brain reserve,” which is defined as the ability to tolerate disease related pathology in the brain without developing clear clinical symptoms or signs (51, 52). This concept of “brain reserve” is typically discussed with respect to cognition, but has also been extended to the motor domain (53). Supporting our speculation, there is some literature to suggest that increased global connectivity can contribute to “brain reserve.” Here, increased global connectivity of the left frontal cortex has been shown to underlie cognitive reserve in dementia (54, 55).

One further consideration is whether tDCS is the optimal non-invasive brain stimulation technique for facilitating plasticity in brain tumor patients. Transcranial magnetic stimulation (TMS) is used frequently in glioma patients, typically with the goal of mapping eloquent cortex in the preoperative period (56, 57). TMS has a different mechanism of action, working to induce current in the brain via a rapidly changing magnetic field (58). TMS has the benefit of directly eliciting action potentials, allowing for quantifiable electrophysiological measurements of motor evoked potentials. When delivered with repetitive pulses (rTMS), the technique can modulate cortical plasticity and enhance motor performance in healthy (59) and patient populations (60). Whether or not rTMS can facilitate plasticity in glioma patients remains to be determined. Further, while there is extensive use of single pulse navigated TMS in glioma patients, the safety profile of rTMS in this patient population has not been investigated.

There are important limitations to this study that must be considered. Firstly, no conclusion can be made about the relative influence of tDCS or motor training on the connectivity results. Indeed, these results may represent an effect of motor training alone, though this also cannot be concluded from our data. Motor training has been shown to modulate connectivity in the sensorimotor network independent of tDCS (61), though tDCS can facilitate motor learning (13), and can affect motor connectivity independent of training (62). The appropriate conclusion to be drawn from the current study is that sensorimotor network connectivity likely changed as a result of the applied intervention, without specifying between tDCS or motor training. This study was designed as an open-label, proof of concept pilot trial aimed at recruiting a group of glioma patients in order to demonstrate that a tDCS/motor training intervention is feasible in this patient population. We recruited a relatively homogenous group (based on tumor location on the left and in proximity to the central sulcus) to explore if the sensorimotor network can undergo changes with a preoperative intervention. As such, the sample size is small, and the findings must be considered in light of this. Further, the clinical implications of these functional connectivity changes are unclear. Previous work has suggested connectivity of the sensorimotor network can track motor ability in glioma patients, with increased connectivity corresponding to increased strength (31). However, as discussed, it is unclear if increased connectivity bestows motor reserve. Importantly, the lack of a control group precludes any definitive conclusions about the effect of our intervention. However, we believe this experience is worth reporting given its high novelty and the potential to spur further investigation into this understudied patient population. We have outlined many of the unresolved questions and areas of future research in Table 3.

**Table 3:** Future directions for clinical translation.

1	What is the optimal motor training paradigm for facilitating plasticity of motor networks in glioma patients?
2	What are the optimal electrode configurations and stimulation parameters?
3	What is the minimum length of time for combined motor training and NIBS to induce long-lasting changes in motor networks?
4	Does facilitating plasticity in the preoperative period lead to improved extent of resection?
5	Does facilitating plasticity in the preoperative period lead to improved motor outcomes following surgery?
6	How does the tumor affect the electric field magnitude within the brain?
7	Are there dose-response relationships between electric field magnitude and motor network plasticity?
8	What is the best measurement of network reorganization in glioma patients?
9	How does glioma grade and genetics alter response to NIBS?

*NIBS, non-invasive brain stimulation*.

## Conclusion

In summary, we demonstrate that tDCS is feasible in preoperative glioma subjects, and that preoperative motor training combined with tDCS may alter sensorimotor network connectivity. Patient specific modeling of the electrical field suggests there may be a dose-dependent relationship between stimulation and connectivity changes. Given the difficult problem of eloquent cortex tumors, novel and innovative strategies need to be designed to benefit patients who otherwise have limited treatment options. Our results suggest that the possibility of modulating neural networks prior to surgery in order to confer resiliency against impending insult is possible. Further work needs to be done to determine how long these changes last for, the optimal training and stimulation paradigms, including any dose-dependent effects, and whether or not modulating the functional connectivity of networks has any clinical benefit for patients. Overall, this proof of principle pilot trial is, to our knowledge, the first study attempting tDCS in glioma patients and supports future investigations into neuromodulation for patients with brain tumors.

## Data Availability Statement

The raw data supporting the conclusions of this article will be made available by the authors, without undue reservation.

## Ethics Statement

The studies involving human participants were reviewed and approved by Conjoint Health Research Ethics Board of the University of Calgary. The patients/participants provided their written informed consent to participate in this study.

## Author Contributions

SL: conceptualization, methodology, investigation, formal analysis, writing-original draft, writing-review and editing, and visualization. LG: methodology, investigation, and writing-review and editing. CM: investigation. AK: conceptualization and writing-review and editing. OM and JK: writing-review and editing and supervision. All authors contributed to the article and approved the submitted version.

## Conflict of Interest

The authors declare that the research was conducted in the absence of any commercial or financial relationships that could be construed as a potential conflict of interest.

## Abbreviations

tDCS: transcranial direct current stimulation
M1: primary motor cortex
EF: electric field
ROI: region of interest
IC: intrinsic (global) connectivity
LCOR: integrated local correlation.
