# Supplementary material for: Preoperative Transcranial Direct Current Stimulation in Glioma Patients: A Proof of Concept Pilot Study
Source: Front Neurol. 2020 Nov 19;11:593950. doi: 10.3389/fneur.2020.593950 (PMC7710969; doi:10.3389/fneur.2020.593950)
Supplement: Supplementary file 1 [file Table_1.DOCX]

**Preoperative transcranial direct current stimulation in glioma patients: a proof of concept pilot study**

Stefan Lang PhD, MD^1,2,3^, Liu Shi Gan PhD^1,2,3^, Cael McLennan^1^, Adam Kirton MSc, MD^1,2,3^, Oury Monchi PhD^1,2,3^, John J.P. Kelly PhD, MD^1,2,4^

^1^Hotchkiss Brain Institute, University of Calgary, Calgary AB, Canada

^2^Department of Clinical Neurosciences, University of Calgary, Calgary AB, Canada

^3^Non-invasive Neurostimulation Network, University of Calgary, Calgary AB, Canada

^4^Charbonneau Cancer Institute, University of Calgary, Calgary AB, Canada

**Supplementary Table 1**

| **Subject** | **Left MNI Coordinates (x,y,z)** | **Right MNI Coordinates (x,y,z)** |
| --- | --- | --- |
| **Patient** |  |  |
| 1 | -46 -18 53 | 40 -22 51 |
| 2 | -32 -20 58 | 44 -25 56 |
| 3 | -32 -39 54 | 39 -19 45 |
| 4 | -38 -19 61 | 33 -21 47 |
| 5 | -40 -23 58 | 39 -23 53 |
| 6 | -37 -20 67 | 41 -22 63 |
| 7 | -40 -30 63 | 36 -22 59 |
| 8 | -29 -37 65 | 33 -23 61 |

MNI coordinates for center of individualized 6 mm M1 seeds. Coordinates based on peak activation voxel from unilateral task fMRI.
